# Portable lensless wide-field microscopy imaging platform based on digital inline holography and multi-frame pixel super-resolution

**DOI:** 10.1038/lsa.2015.119

**Published:** 2015-10-23

**Authors:** Antonio C Sobieranski, Fatih Inci, H Cumhur Tekin, Mehmet Yuksekkaya, Eros Comunello, Daniel Cobra, Aldo von Wangenheim, Utkan Demirci

**Affiliations:** 1 Demirci Bio-Acoustic-MEMS in Medicine (BAMM) Laboratory, Division of Biomedical Engineering, Division of Renal Medicine, Department of Medicine, Brigham and Women’s Hospital, Harvard Medical School, Boston, MA, USA; 2 Demirci Bio-Acoustic-MEMS in Medicine (BAMM) Laboratory, Division of Infectious Diseases, Brigham and Women’s Hospital, Harvard Medical School, Boston, MA, USA; 3 INCoD - National Brazilian Institute for Digital Convergence/LAPIX, Image Processing and Computer Graphics Lab, Federal University of Santa Catarina, Brazil; 4 Demirci Bio-Acoustic-MEMS in Medicine (BAMM) Laboratory, Stanford University School of Medicine, Canary Center at Stanford for Cancer Early Detection, Palo Alto, CA, USA; 6 4VisionLab, Master in Applied Computing, University of Itajaí Valley, Brazil; 7 CERTI Foundation, Florianopolis, Brazil

**Keywords:** digital in-line holography, image registration, lensless imaging, point-of-care platform, pixel super-resolution

## Abstract

In this paper, an irregular displacement-based lensless wide-field microscopy imaging platform is presented by combining digital in-line holography and computational pixel super-resolution using multi-frame processing. The samples are illuminated by a nearly coherent illumination system, where the hologram shadows are projected into a complementary metal-oxide semiconductor-based imaging sensor. To increase the resolution, a multi-frame pixel resolution approach is employed to produce a single holographic image from multiple frame observations of the scene, with small planar displacements. Displacements are resolved by a hybrid approach: (i) alignment of the LR images by a fast feature-based registration method, and (ii) fine adjustment of the sub-pixel information using a continuous optimization approach designed to find the global optimum solution. Numerical method for phase-retrieval is applied to decode the signal and reconstruct the morphological details of the analyzed sample. The presented approach was evaluated with various biological samples including sperm and platelets, whose dimensions are in the order of a few microns. The obtained results demonstrate a spatial resolution of 1.55 µm on a field-of-view of ≈30 mm^2^.

## INTRODUCTION

Microscale imaging in life sciences so far has been associated with conventional light microscopes using geometric optic^1^. The advent of the holography principle, in 1948 by Dennis Gabor^2^, allowed the development of electron microscopes with high-resolution (HR), opening a wide range of investigation for both microscale and nanoscale microscopy. Such optical instruments, however, may require advanced infrastructure, being mostly restricted to well-established institutes. Particularly, in resource-limited or in developing countries, inexpensive, reliable, and user-friendly imaging devices are critical for the diagnosis and treatment of a series of diseases^3–12^.

With recent advances in opto-electronic technologies, miniaturized lensless imaging devices for point-of-care (POC) platforms have been developed, where the shadow imaging principle is commonly utilized in portable devices. Shadow imaging is a simple technique, whose working principle is to illuminate the specimens and capture two-dimensional (2D) spatial signatures (shadows) with an electronic image detector placed underneath^13,14^. Interestingly, by using a coherent light-source (*i.e.*, monochromatic wave), the spatial signatures are converted to pattern interferences of the analyzed sample, and holographic information is obtained according to the scalar diffraction theory. By utilizing this pattern, numerical methods for phase-retrieval can be used to reconstruct the morphological details of the sample at specific object-planes.

From a technological perspective, a portable lensless holographic platform presents some advantages compared to conventional optical microscopes. The first aspect is the simplicity and cost, where the entire system can be built in a few cubic centimeters, using off-the-shelf components such as pinholes, monochromatic light-emitting diodes (LEDs) and complementary metal-oxide semiconductor (CMOS) and charge-coupled device (CCD). Second, the platform works with no lens requirements, and the investigation of a specimen is performed directly over the imaging sensor surface^13,15–17^, which presents a wide field-of-view (FOV) for high-throughput applications. Third, a single hologram contains all the three-dimensional (3D) structural information of the specimen, and using a high-speed camera, four-dimensional (4D) *in-situ* inspection is also possible to be implemented. Another important aspect related to portable lensless platforms is the easy customization for hardware (*e.g.*, microfluidic) and the integration with embedded software. With the power of the recently developed processor units, many image processing methods can be integrated to perform low-level and high-level procedures, such as decision-making tasks. For low-level applications at the image formation level, resolution can be also improved computationally during the acquisition step. Spatial resolution is particularly determined by the pixel-size of the imaging detector, and some physical limits can be overcome by computing a HR image with a larger density of pixels^18,19^. In the literature, a HR image can be obtained from a single or multiple frames. For a single frame, physical aspects of the sensor should be known in advance and a point-spread-function (PSF) is estimated to convolve the image and improve sharpness information^20,21^. When a coherent illumination system is used, however, as the image formation is linear in the complex field, the estimated PSF function can produce some cancellations or other undesirable non-linear effects^22^. For multiple frames, a higher-resolution image can be obtained from consecutive frames taken over time^23–25^. This improvement of resolution is possible since physical variations are introduced in time, such as pixel shifts or different flickering intensities of light achieving the imaging sensor cells.

In this paper, a portable lensless wide-field holographic platform based on irregular shifts of the light-source is presented. This approach combines digital inline holography (DIH) and multi-frame pixel resolution approaches to compute a single higher-resolution holographic image from a set of observations of the scene, obtained from small planar displacements of the light-source. A registration procedure is performed to align the multiple images onto the same planar domain, and then, a sub-pixel optimization approach is used to minimize a cost-function designed to increase the quality of the holographic information. Finally, a numerical phase-retrieval method is used to recover the morphological information of the corresponding sample at specific object-planes. The presented portable platform was evaluated considering static circular and non-circular human cells in the order of a few microns, such as sperm and platelets. The results also show a spatial resolution of 1.55 µm on a maximum FOV of ≈30 mm^2^.

## MATERIALS AND METHODS

### Experimental setup

**Figure 1:**
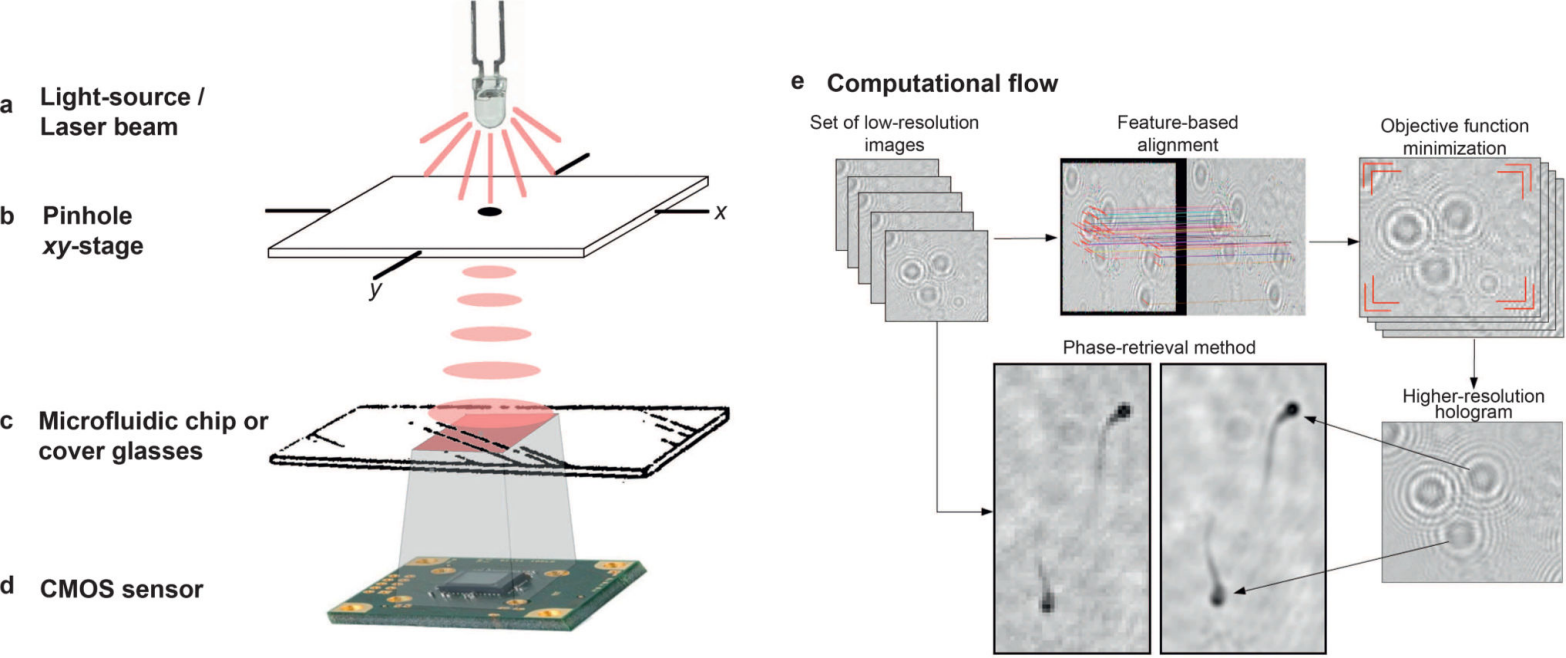
Schematic representation of the experimental setup used to acquire holographic signatures. In (**a**) the light source is used with a pinhole placed over, as shown in (**b**). In (**c**) the object-plane corresponding to the sample to be inspected is illustrated. The detector-plane is shown in (**d**), where a CMOS-based imaging sensor is used. (**e**) General overview of the presented approach: set of LR images with small shifts of the light-source is registered and an optimization method minimizes an objective function. The obtained HR hologram is then processed by a phase-retrieval method into morphological information. Noise suppression can also be obtained from the multiple shifted images.

An experimental setup was developed to acquire holograms and produce shifts of the light-source. This setup and its components are demonstrated in Figure 1a–1d: (a) light-source, (b) spherical aperture and *xy*-stage built to displace the light-source, (c) surface to accommodate the sample, and (d) the imaging sensor. In Figure 1a, a light-source is used to illuminate the samples with a pinhole (b), where an ultraviolet LED of 385 nm, model M385L2 from ThorLabs was used. To produce the *xy*-stage demonstrated in Figure 1b, a CO_2_ laser cutter engraver model Versa Laser 2.3 was used. This stage has a chamber to scatter the light and two independent platforms on the top specifically designed to displace the light-source on *x*- and *y*-axis directions independently. This chamber was projected to image samples using cover glasses or microfluidic channels (Figure 1c), and signatures are recorded by a CMOS sensor (Figure 1d) and a *xy*-stage having dimensions of 10 × 10 cm^2^ placed 15 cm away from the sensor. On top of it, LED was placed in a cylindrical structure with 100 mm diameter pinhole located 15 cm away from the sensor. CMOS imaging sensor is fixed from the bottom side of the platform, where the distance between sample and electronic detector is as small as possible. The CMOS sensor used to capture the hologram shadows is an Aptina 10 megapixel monochromatic sensor with dimensions of 6.413 mm × 4.589 mm (≈30 mm^2^), spatial resolution of 3840 × 2748 pixels, frame rate of 3.2 frames per second, and 1.67 µm of pixel-size. 8-bit raw data can be directly accessed from the sensor *via* Software Development Kit, providing a practical manner to implement data acquisition, numerical diffraction and image analysis on the same portable platform.

One of the advantages of our portable holographic platform for microscopy analysis is simplicity and low cost, since no lenses are required to image samples. The presented holographic platform can be built using in expensive materials, such as UV light-source for illumination, pinholes, and the setup can be designed in a conventional laser cutter engraver to accommodate component parts. The most relevant component is the CMOS imaging-based detector, which meets the requirements (monochrome and reduced pixel-size) to compute pixel information, and costs 500 US dollars. The total cost of the presented portable platform using a single light-source is estimated in less than 600 US dollars with LED, pinhole and home-made PMMA parts.

Using the experimental setup described, a set of LR images is obtained from a displacement matrix of varied sifts of the light-source (7 × 7 different positions). To improve resolution, a two-step registration procedure is used to (i) align the LR images on the same planar domain by a feature-based method and (ii) optimize sub-pixel information by the minimization of an objective function. The registration method keeps the initial pre-alignment points from a feature-based registration method, while seeking for sharpness optimization in a pixel level. Thus, we obtain a HR image where the propagation of the holographic signal are improved by recover the missing information from the set of LR images, as illustrated in Figure 1e.

### Feature-based image registration

Image registration is the process of computing an optimal transformation between two or more images and align them on the same domain. The pre-alignment procedure is a feature-based registration method performed as follows^26^:

- The key-points are detected for the both reference and candidate images, and a feature-vector is calculated. The Speeded Up Robust Features (SURF) algorithm^27^, based on the sums of Haar wavelets and inspired on the Scale-Invariant Feature-Transform (SIFT) algorithm, is used to locate the relevant key-points^26^.
- The feature-vectors of the reference and candidate images are matched by a neighborhood function. The Fast Approximate Nearest Neighbor Matcher was used to correlate key-points^28^.
- To select only high-quality matches, the obtained results are validated by a clustering technique designed to remove outliers’ points. These outliers can be detected by a least-square fitting approach based on the geometrical distances and angular coefficients between reference and candidate images of the LR set^26^.
- The resulting matrix-matching is computed for every single LR image using the Random Sample Consensus algorithm^29^.

Feature-based methods, on the other hand, are not properly designed for the sub-pixel alignment and interpolation once key-points represent an approximate matching of features. To optimize the alignment in a sub-pixel level, the homography matrix is used as an initial guess of the global optimum solution and an area-matching method is used to optimize alignment, as detailed in the next section.

### Sub-pixel optimization using continuous ant colony optimization (ACO_ℜ_)

In this approach, sub-pixel optimization is performed by the ACO_ℜ_ algorithm^30^. Differently from the traditional ACO algorithm, designed to be a metaheuristic for discrete combinatorial problems, the ACO_ℜ_ is a category of algorithms for continuous optimization. As a typical formulation of combinatorial problems, continuous optimization is determined by a search-space *S* designed to operate over a set of decision variables and an objective function *f*: *S* → ℜ_0_^+^ to be minimized. *S* is a set of continuous variables *X_i_*, *i* = 1, …, *n*, and it has the constrained or unconstrained variables, *i.e*., *v_i_* ∈*D* domain. A solution s* ∈ *S* is the best configuration of the continuous variables (i.e., for the sub-pixel registration problem) and it is considered a global optimum solution (the best minimization of an error metric) if and only if *f*(s*) ≼ *f*(*s*) for each possible *f*(*s*) ∈ *S*. The set of all global optimum solutions is presented by S* ⊆ *S*, where at least one s* should be found to solve the continuous optimization problem. As we can see, this is an iterative process, where better solutions (with a minimum error) are kept to substitute worst ones in a minimization procedure.

To find s*, the minimization of an energy cost-function based on variational models is used for the holographic sub-pixel problem^31,32^. Two penalizer terms were used in our cost-function: a data term and a sharpness measure. The first term (i.e., data or fidelity term) penalizes deviations of a candidate HR image from the set of multiple LR images, expressing the assumption that the result should be close to the data during the minimization. The second term is a sharpness measure designed to indicate when a candidate solution is producing better holographic propagation. Consider a candidate solution containing the LR images transformed by a matrix-matching, associated with a HR image *I*. The energy *E* of this solution is defined as:

(1) $$E(I,L)=\alpha \sum_{i=1}^{m}{\left(L_{i}-I\right)}^{2}+\beta (\nabla I)$$

where *L* is the set of transformed LR observations, and *m* is the cardinality of *L*. The aforementioned equation is similar to the general formulation for super-resolution presented by the Ref. 33, but with the addition of a term specifically designed for holographic propagation. First term is the fidelity term measuring the quality of approximation between each LR image in *L* to *I*, being related to spatial locality, penalizing solutions whose correspondence to *I* is inappropriate. (i.e., large E). The second term *I* is a focus measure used to compute the relative focus degree of an image, and can be obtained from the sum-modified-Laplacian method (LAPM)^34^. Parameters *α* and *β*, like in many variational models formulated over the literature^31^, controls the relative importance for each penalizer term. As the LR set is obtained upon spatial displacements (shifts of the light-source), some diffraction patterns may change according to the position of the light-source, shape and height of object-plane, and holographic fringes may be slightly different for each LR image. Feature-based alignment and sub-pixel optimization computational procedures are also illustrated in Supplementary Fig. S1.

### Diffraction calculation

**Figure 2:**
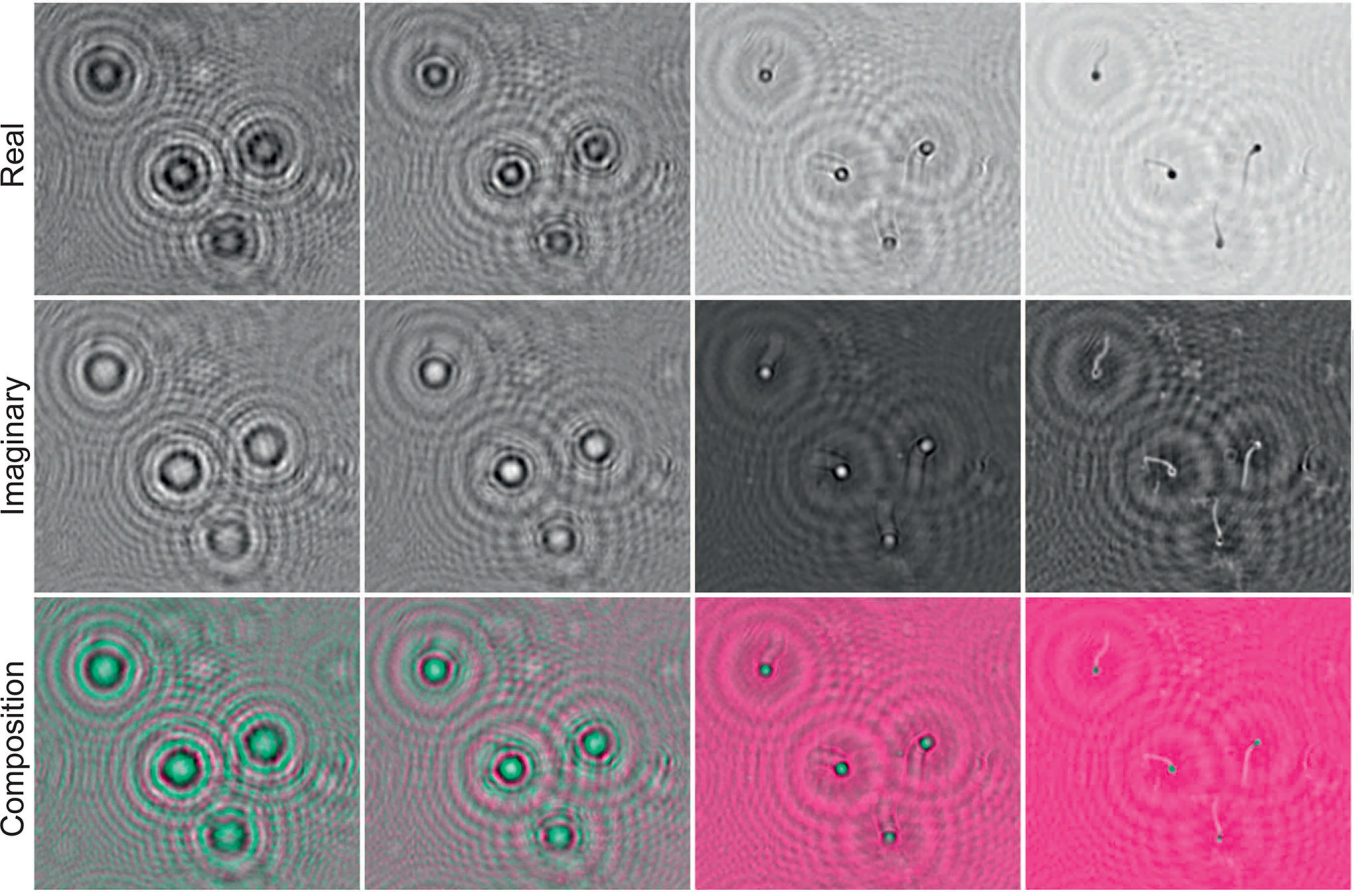
Numerical diffraction of a sperm sample hologram. First to third rows the real (ℜ), imaginary (ℑ) and composition (ℜ + ℑ) parts of the diffraction are shown, respectively. Diffractive distances from the first to fourth columns are *z* = [30k, 45k, 60k, 70k] (*k* = ×1000), according to Equation (S3) of Supplementary Information.

Diffraction calculation is a computational numerical solution to the phase problem when a wavefront intercepts openings and obstacles. In Figure 2, the diffraction computed by the Angular Spectrum Method (ASM) applied to holograms of sperm sample is presented. From the first to third rows, the real (ℜ), imaginary (ℑ) and composition signal are illustrated. Composition is obtained by computing a multidimensional signal from *I*. Using a composition signal, we visualize sperm heads and sperm tails with distinct color intensities, and then, allow automatic counting and image analysis. By varying *z*-specific object-planes are obtained, and distinct *z*-axis information can be visualized. Details in diffraction calculations were stated in Supplementary Information.

### Biological sample preparation

Biological human samples were used to validate the presented experimental setup and algorithmic solution. Sperm refers to the male reproductive cells, with dimensions at the order of 3–5 µm^35^, and 55 mm long tail with a diameter of 0.5 µm^36^. Sperm cells are particularly interesting, presenting unique characteristics and morphology, so they can be easily identified using the presented approach even without microscopy confirmation. Sperm samples were in liquid nitrogen. For sampling, we thawed sperm at 37°C, and diluted in 1% bovine serum albumin (BSA) in human tubulin fluid media (1:20 dilution with the media). For the analysis, 10 µL was added on a glass slide, and mounted with an 8 × 8 mm cover slip. Sperm samples were incubated on the glass slides until they are metabolically dead, and thus, we evaluated sperm morphology in static mode.

Platelets, on the other hand, are small disc-shaped cell fragments with greatest diameter varying from 2 to 3 µm^37^, playing significant roles to repair and regenerate connective tissues. For evaluation of platelet morphology, platelet cells were captured from whole blood in microchannels. To produce microchips with nine-microchannels, poly(methyl methacrylate) (PMMA) (40.5 × 24.5 mm), double-sided adhesive film (DSA, 50 µm for microchannel height) and glass cover slide (40.5 × 24.5 mm) were used^38^. The inlets and outlets (0.65 mm in diameter, 26 mm apart) were cut on the PMMA layer using a VersaLASER (Universal Laser Systems Inc., Scottsdale, AZ, USA). DSA was cut to form the microchannels (6.6 × 2.4 × 0.05 mm) using the same procedure. This DSA layer was then attached to the PMMA layer. A glass slide was modified with oxygen plasma (100 mW, 1% oxygen) for 90 seconds in a PX-250 chamber (March instruments, Concord, MA, USA), followed by silanization step using 200 mM of 3-mercaptopropyl-trimethoxysilane (3-MPS, Sigma-Aldrich, St. Louis, MO, USA) dissolved in ethanol. After the silanization step, the glass slide was assembled with the PMMA-DSA. 2 mM of N-*γ*-maleimidobutyryl-oxysuccinimide ester (GMBS, Sigma-Aldrich, St. Louis, MO, USA) in ethanol was used as a cross linker of NeutrAvidin (Thermo Fisher Scientific Inc., Waltham, MA, USA) binding, followed by antibody immobilization (CD62p antibody biotin anti-human, Biolegends, San Diego, CA, USA) on NeutrAvidin-modified surface^39^. After surface chemistry steps were completed, whole blood which was diluted 1:40 in a buffer with 5% acid-citrate-dextrose, 2% Tyrode’s buffer, 3% Apyrase from potatoes, 5% BSA, 1% HEPES, in phosphate-buffered saline (PBS) was introduced into microchannels. After 20 minutes of incubation time PBS applied into microchannels to remove other blood cells and plasma and captured platelets are ready for observing. Further, we used a fluorescent-labeled antibody (Mouse Anti-human CD41: Alexa Fluor® 647 antibody, AbDSerotec, Raleigh, NC, USA) to visualize the captured platelets under microscope.

Samples were imaged using the presented approach, and microscopy analysis was also performed. To acquire the set of LR images, static samples were considered. To conduct our experiments, a microscopy analysis was performed using an inverted microscope Carl Zeiss Axio Observer-Z1. For sperm and platelet samples, microscope mosaic images with 10× were obtained. Bright-field images were acquired for both sperm and platelet samples for visual comparison. Additionally, fluorescent microscopy images were acquired for confirmation of platelets after incubating with Cy-5-labeled detection antibodies.

## RESULTS AND DISCUSSION

### Resolution improvements for holographic signatures

**Figure 3:**
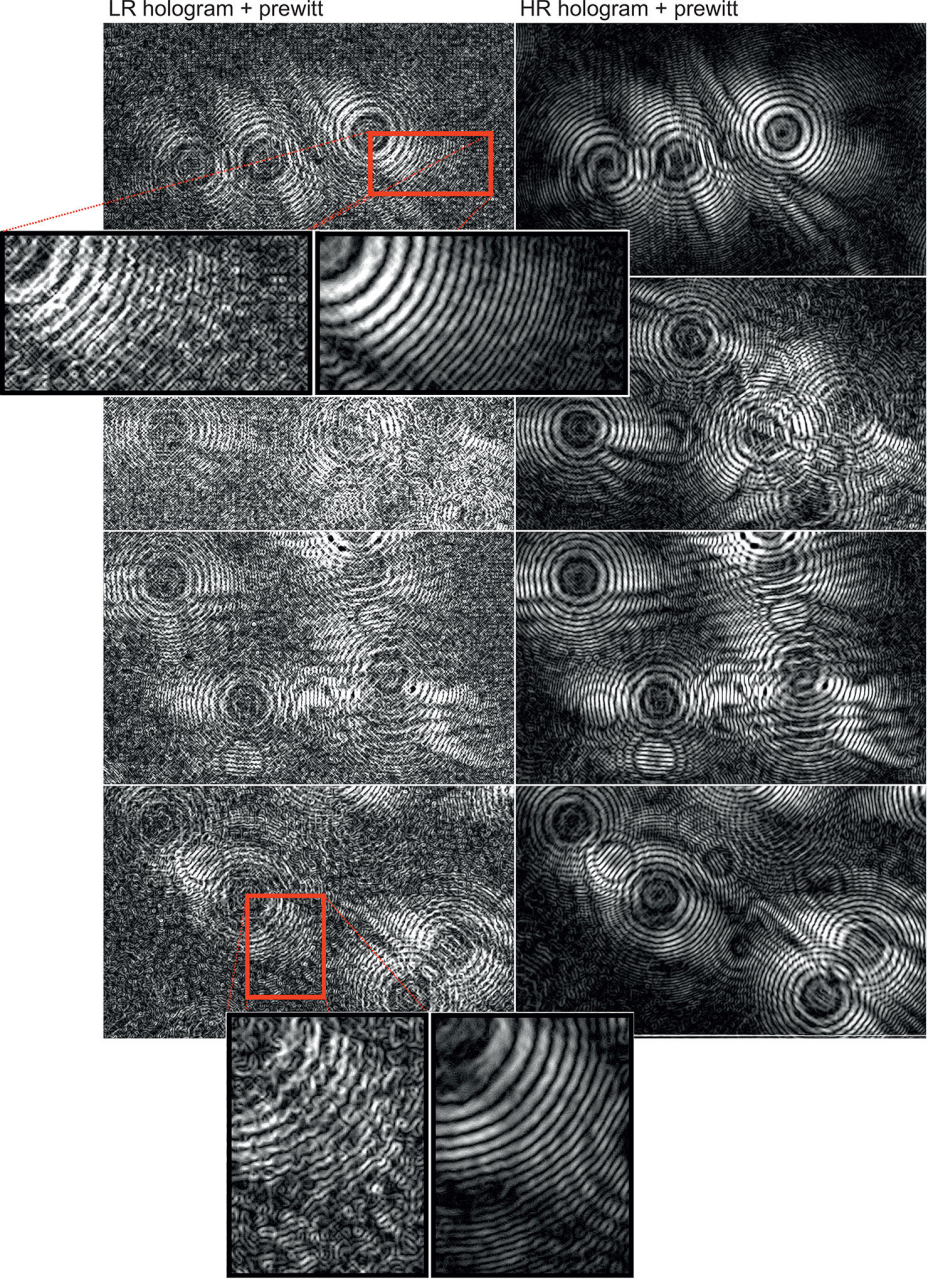
LR and HR holograms of sperm samples. The prewitt compass is used for visualization purposes. At the left side, a single-shot (*i.e*., LR) holographic image is presented, and at the right side, the holograms are combined into a HR image obtained from 49 observations of the scene.

A notable aspect to be considered in terms of qualitative and quantitative analysis is related to the quality of the holographic signatures obtained on the registered images. Low and high frequencies are fundamental in holography, and the number of cycles defines the level of details recovered by a numerical diffraction method. In Figure 3, the results are demonstrated using the sub-pixel registration method. First and second column correspond to LR and recovered HR holographic images, respectively. Holographic images were submitted to the Prewitt compass to make visible specific details of the reconstruction procedure, once they may not be perceptible for raw holographic images. Prewitt compass is a discrete differentiation operator used to compute the approximation of the gradient related to the image intensity, and it is better understood as either the gradient vector or the vector-norm for each coordinate in the input image. The results suggest two main aspects to be observed regarding resolution and noise suppression. Qualitative improvement of holographic frequency can be clearly observed by the number of recovered fringes and the quality of morphological information, as well as the effect of noise suppression for background areas.

**Figure 4:**
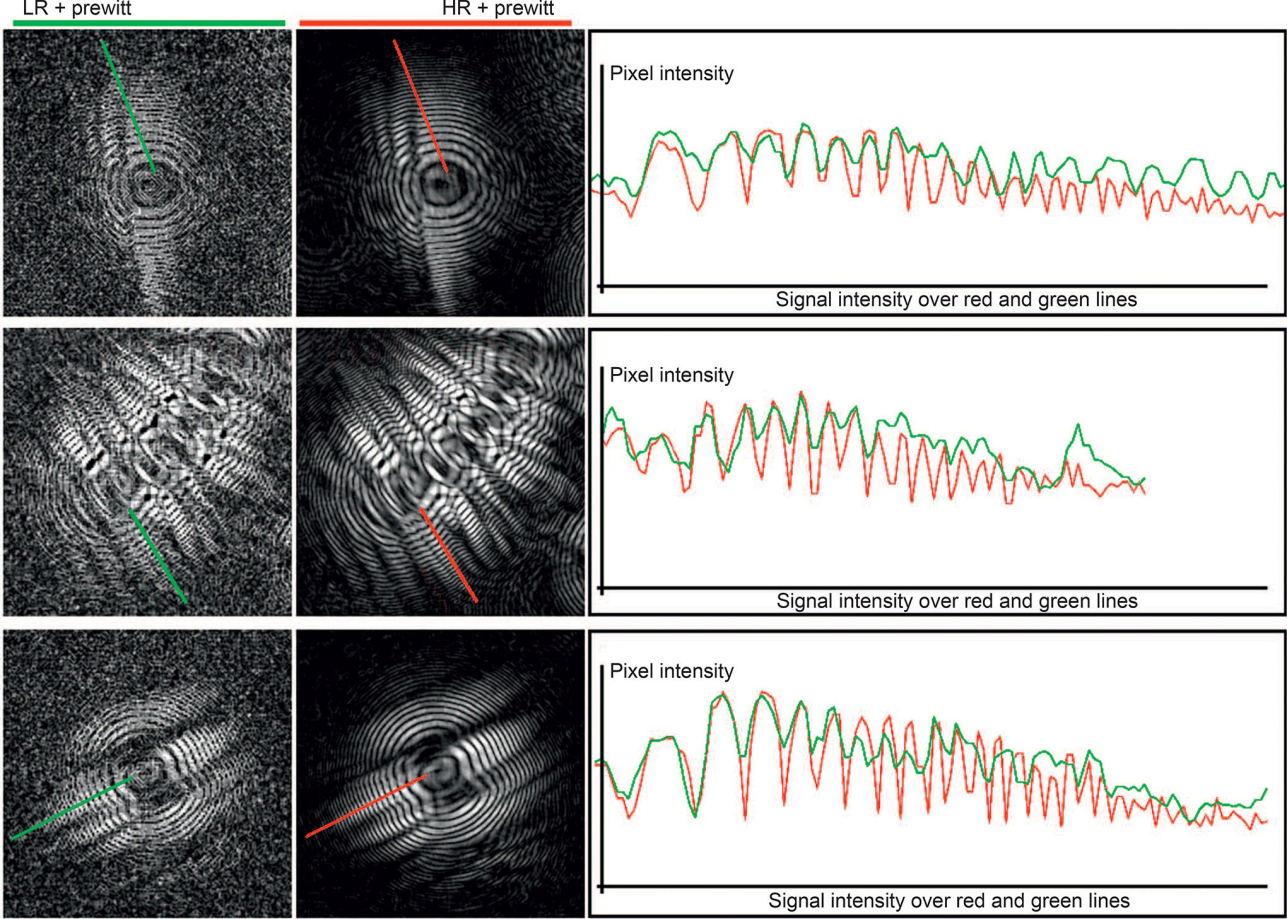
Quantitative evaluation of LR and reconstructed HR holograms. LR and HR holograms are shown at the left and right side, respectively. A measure of the intensities was performed over the red and green lines drawn on the LR and HR resolution hologram, respectively. At the right side, these intensities were plotted, showing a better propagation and signal discrimination for the HR holographic images.

In Figure 4, a quantitative evaluation of the number of cycles for LR and HR holograms was performed. At the left and right side, the LR and HR holograms are presented, followed by the periodic analysis of holographic signal over a specific area (right side). The measurements are defined by the green and red lines over the LR and HR images, respectively. For the HR holograms, where the morphological information is improved and the high frequencies recovered, the number of gradient peaks is better when compared to those presented in a LR hologram, captured from a single frame. Generally, LR holograms present periodicity until the seventh or eighth cycle, although intensities are also well-described by the HR holograms. For a higher number of cycles, the correspondence between LR and HR is lost, suggesting better quantitative results for the multi-frame approach.

In addition, noise may affect the quality of signal for the LR images, especially for high frequencies. Noise can be introduced by physical aspects, such as oscillatory differences of the light intensities captured by the sensor cells over time. In the Figures 3 and 4, some of the mentioned variations can be verified for the LR images, where several gradient peaks can be visualized along the background areas. Undesirable physical artifacts such as dust or scratches that may be introduced on the sensor surface or the top of the microfluidic or cover glasses can be minimized during the acquisition by the presented platform. These artifacts, which are not located at the same object-plane, are suppressed and redistributed on the HR holographic image. The artifact suppression is possible with no additional hardware or software computation thanks to the diffraction of light, which is differentiable for each object-plane when the point-source is changed. The displacement vector for such artifacts does not match with the vectors located at the same object-plane, and thus the matrix-matching ensures the spatial distribution of these false-positive particles.

### Evaluation and microscopy analysis

**Figure 5:**
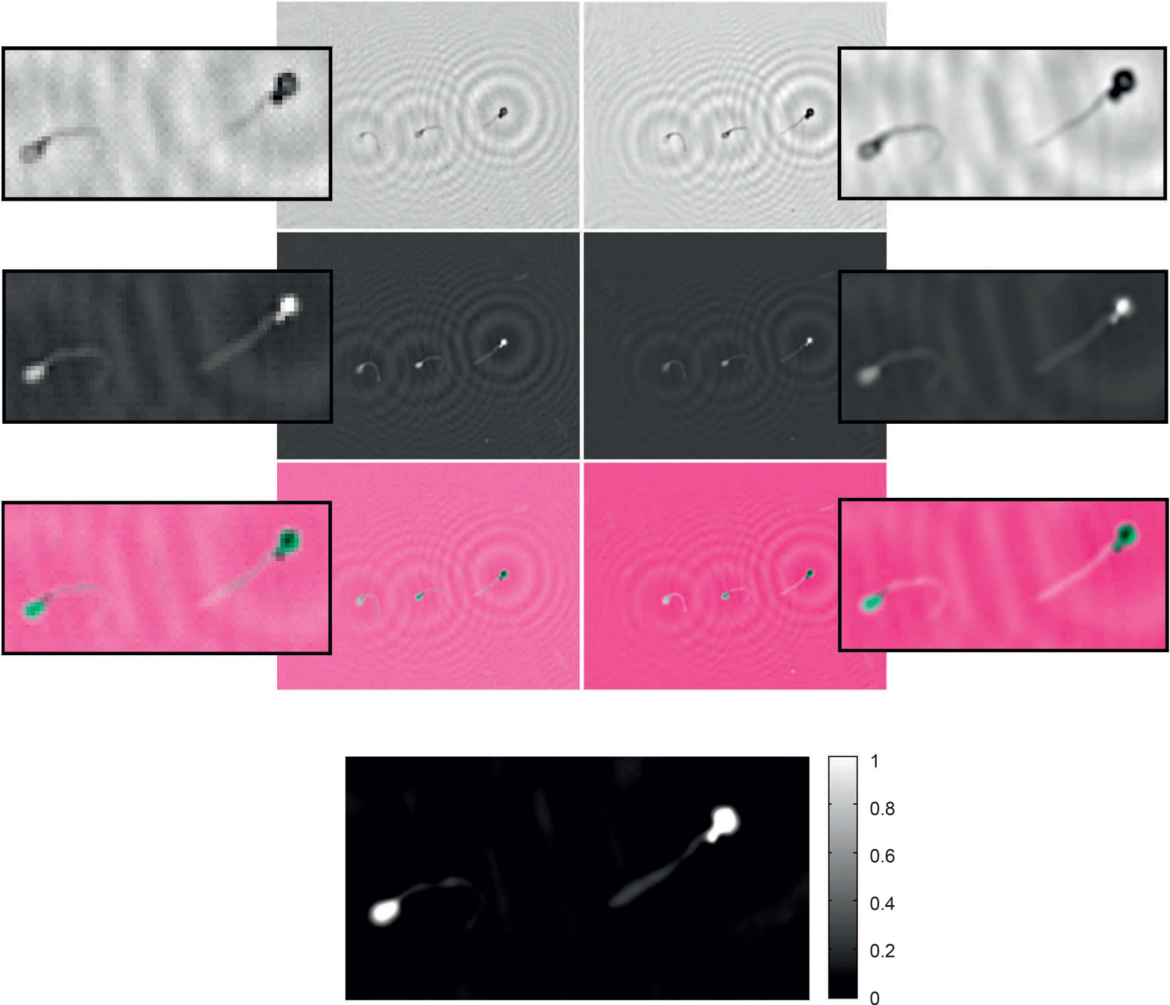
Comparison of reconstructed LR (left side) and HR (right side) holograms, recovered from holograms shown in Figure 3 (first row holograms). From the first to third rows, the amplitude, phase, and multi-dimensional RGB composition signals are shown, respectively. Last row shows the normalized HR phase in radians, having values corresponding to (min. = −1.43, max. = 3.1416).

**Figure 6:**
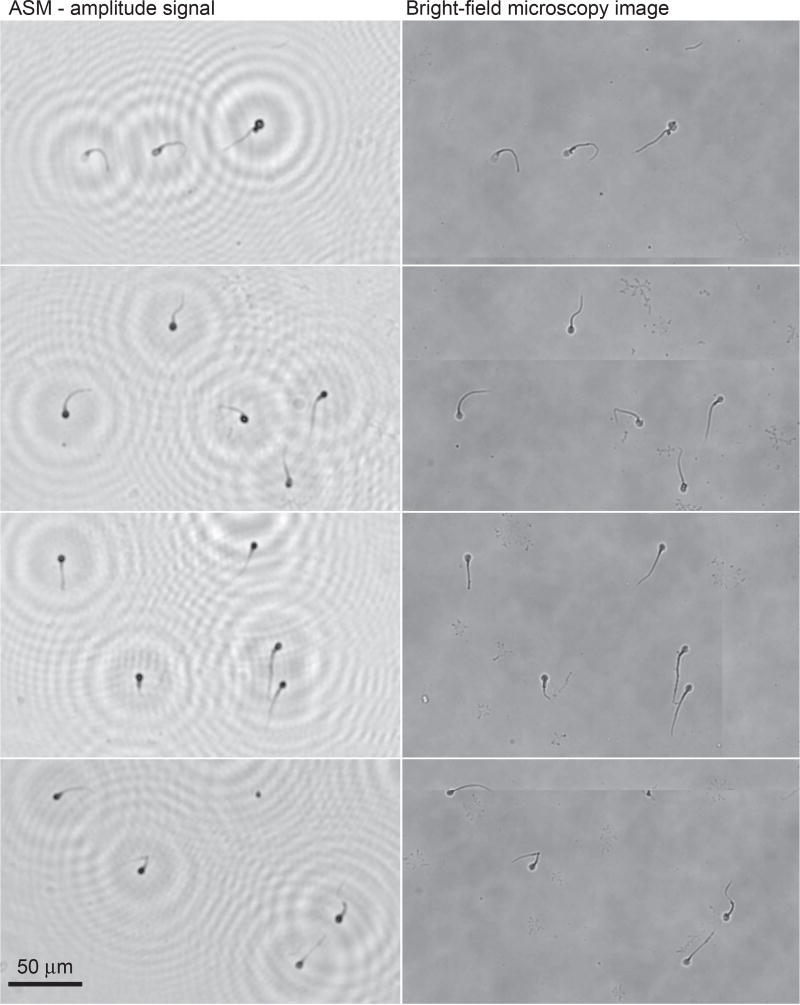
Comparison of the obtained holographic and bright-field microscopy analysis of sperms. Left side are the obtained results for decoded HR holographic images previously presented in Figure 4, and the corresponding bright-field microscopy images are shown at the right side for confirmation purposes.

Microscopy analysis was performed to compare the results of the portable platform. The amplitude signal of the ASM was used for visual comparison against the corresponding bright-field image of the microscope. Holograms of sperm cells presented in Figure 3 (first row holograms) were recovered by the ASM, as shown in Figure 5, where LR and HR results were presented for single shots and HR sub-pixel optimization, respectively. Amplitude, phase, and composition of the RGB signal were obtained. In HR images, improved resolution and noise suppression for the background areas was observed when compared to LR images. A higher level of high-frequency signal allows better level of details for numerical reconstruction. In Figure 6, the reconstruction of the HR holograms previously shown in Figure 3 is demonstrated with the corresponding bright-field microscopy image. A visual comparison demonstrates a high correlation of the obtained results and the bright-field microscopy image, even for small morphological structures, such as sperm tails.

**Figure 7:**
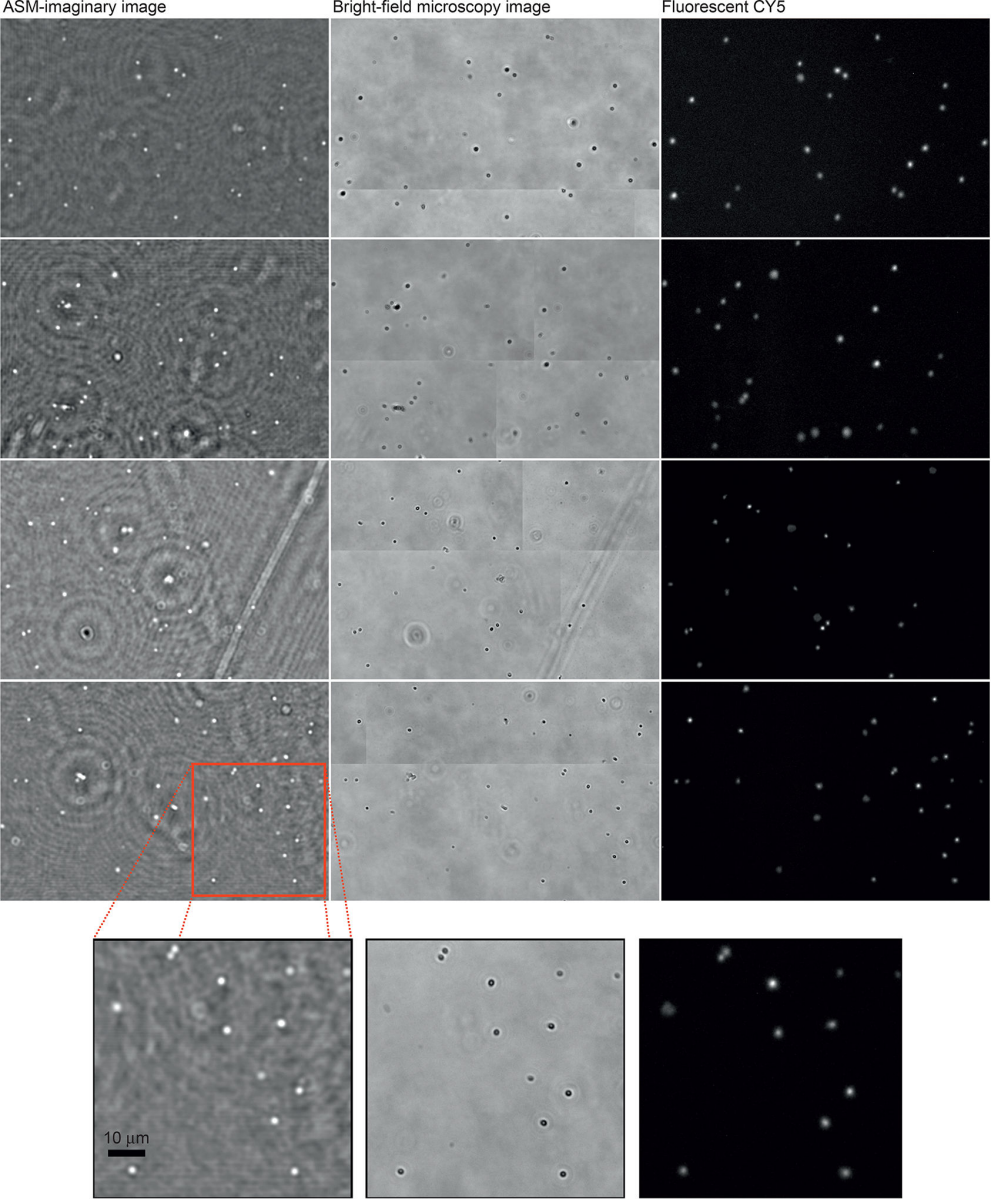
High-resolution platelet holograms recovered by ASM. From left side to right: decoded platelet image from ASM, bright-field microscopy image, and fluorescent microscopy image, respectively. At the bottom side, zoomed areas are shown.

Platelet cells (2–3 µm in size) were also imaged in the platform, as shown in Figure 7. The result of the numerical diffraction was shown in the first column, where the ℑ-part is presented. ℑ-part is particularly interesting especially when acquired holographic shadows are weak, providing better contrast responses. Microscopy confirmation was performed using the bright-field (middle), and Cy5 fluorescent microscopy images were also presented at right side of Figure 7. A high correlation can be also observed for both presented approach and microscopy analysis.

## CONCLUSIONS

Lensless imaging technologies present great impact on the development of portable POC platforms for resource-limited settings, and by associating them with computational pattern recognition and digital image-processing techniques, novel platform technologies are developed for real world applications. The presented portable lensless wide-field microscopy platform is one example for such applications, where both imaging sensors and computational image interpretation are integrated on the same platform for micron analysis. This platform is also validated with multiple imaging applications, such as the visualization of sperm morphology and the counting of platelets on-chip. Thus, we provide a new insight to broad applications for POC imaging applications in the future.

The principle of holographic imaging invented many decades ago has significantly advanced since the original digital in-line holographic experiment^2,40^. In the literature, there is a significant number of approaches developed upon the traditional holography principle, where a splitter is commonly used to separate object and reference beams^41–44^. Although traditional holography may presents some limitations regarding miniaturization into POC applications, many of their diffraction concepts can be brought to DIH. Due to its simplicity, DIH is preferable for POC applications, and it can be easily implemented into a miniaturized platform using inexpensive components.^45–57^. Many DIH approaches for POC applications are dedicated to improve holographic signatures by making use of high-speed frame-rate cameras in the order of thousand frames per second (fps)^48–50^. Small sub-pixel sweeping perspectives are then computed, and very slight differences of the sample in motion are recorded over time. Although the elevated acquisition cost for a higher-speed camera, this category of portable platforms can achieve very efficient sub-micron resolutions.

Using a different principle to increase resolution on a large FOV (24 mm^2^) of a conventional imaging sensor, an array of LEDs and a micro-controlled device was used to alternate the light point-source and shift holographic signatures on the sensor surface^51,52^. A HR holographic image is obtained from a set of 23 LR images, and an interactive phase-retrieval method recovers sub-micron scale visualization after a number of iterations is achieved. Previously a single light-source was used to capture sperm holograms to compute their movement with in a rate of 2 frames per second, using a subtraction method between consecutive frames^53^. Similarly, the simulation of LR images captured from different orientations and positions was demonstrated in Ref. 42. Using an array of 25 LEDs connected to fibers, light is scattered to different locations to estimate a HR image. A lensless optical tomographic microscope using a partially coherent in-line holography was presented by Refs. 54 and 55, where a multi-angle lensless imaging platform and tomographic microscope was developed. In this approach the achieved spatial resolution is approximately 1 µm within a sample volume of ≈15 mm^3^. Also, a high-throughput lens-free 3D tracking of human sperms is presented in the Refs. 56 and 57, where two sequential light-sources of different wavelengths were positioned for vertical and oblique planes. Holograms in video are recorded and further decoded to track sperm trajectories in the medium. Recently, a platform was introduced to demonstrate a multi-angle observation model^58^. In the platform, multiple frames were taken from varied perpendicular and angular position, followed by the computation of a higher-resolution hologram. By increasing the distance between object and detector planes, magnification of holograms is obtained. Also, a tunable flexible hexagonal phase grating was used to improve resolution in Ref. 59. The numerical aperture is then increased, improving the spatial resolution for the imaging system.

In our approach, differences can be verified when compared to the aforementioned works in the literature. First, the multiple images are obtained under the illumination of a single and the same light-source and pinhole, arbitrarily displaced (*i.e*., 1–2 mm) over time by a *xy*-stager scheme. A single light-source makes the setup easier and simple to be implemented, and no variations provided by different wavelengths are introduced. Second, resolution for the holographic signal is improved and at the same time noise suppression is performed, as it can be verified in our experimental results section and in supporting reconstruction video. Third, in computational terms, image registration and optimization is performed by both feature-matching and area-based approaches, thus the system is unconstrained to relatively small steps of the light-source. The optimization of the sub-pixel information is performed by energy equations from variational models, and minimized by a bio-inspired framework. Based on this irregular displacement scheme and also computational registration and optimization, the presented platform is operated with no calibration requirements, since there is no need for pre-determined locations of point-source. Our method also tracks holographic shadows dynamically from arbitrary locations (produced by irregular shifts of the light-source), and combines the frames onto the same planar domain to compute a HR frame. The portable lensless wide-field microscopy based on digital in-line holography and multi-frame processing achieves a spatial resolution of 1.55 µm in a maximum FOV of ≈30 mm^2^.

From a computational perspective, multi-frame pixel super-resolution approaches can be greatly used to circumvent physical limits of hardware by recovering the missing holographic information from a set of observations of the scene. In this paper, a two-step registration method was used for both feature-based and area-based registration. The obtained results are closely comparable with those obtained with a conventional microscope using the bright-field.

Further improvements can be performed to increase the efficiency of the presented approach. A stable displacement micro-controlled scheme can reduce the need of the pre-alignment procedure, as well as distortions produced when large displacements are used. Sub-pixel information can be computed with no need of a pre-alignment step once the matrix of displacements for the initial guess solution is static. Artifacts provided by the twin-image problem also should be addressed to remove holographic interference after holograms are processed. On the other hand, intrinsic limitations can be verified related to the Gabor’s inline holography principle, such as twin-image problem and the density of the analyzed sample. Specifically for the confluence of samples, the reference wave gets distorted in the presence of high-density particles, producing self-imposed interferences patterns. Holographic patterns may be deformed due stretching and cross-section of shadows, produced by the incident illumination angle or partial interference in the presence of confluence of high-density objects^60^. Confluence problem can be solved in part by using a very tiny sample or tissue in the order of a few micrometers, where diffractive interference patterns can be captured on the detector plane.

## Supplementary Material

Supplementary Information

Supplementary Video1

Supplementary Video2

Supplementary Video3

Supplementary Video4

Supplementary Video5

Supplementary Video6
