# Supplementary material for: Portable lensless wide-field microscopy imaging platform based on digital inline holography and multi-frame pixel super-resolution
Source: Light Sci Appl. Author manuscript; Available in PMC 2018 Apr 13. (PMC5898403; doi:10.1038/lsa.2015.119)
Supplement: Supplementary Information [file NIHMS925826-supplement-Supplementary_Information.docx]

**Supplementary Information**

**Portable lensless wide-field microscopy imaging platform based on digital inline holography and multi-frame pixel super-resolution**

Antonio C. Sobieranski^1,2,3^, Fatih Inci^4^, H. Cumhur Tekin^4^, Mehmet Yuksekkaya^1,2,5^,

Eros Comunello^3,6^, Daniel Cobra^7^, Aldo von Wangenheim^3^, and Utkan Demirci^1,2,4,*^.

^1^ Demirci Bio-Acoustic-MEMS in Medicine (BAMM) Laboratory, Division of Biomedical Engineering, Division of Renal Medicine, Department of Medicine, Brigham and Women’s Hospital, Harvard Medical School, Boston, MA, USA.

^2^ Demirci Bio-Acoustic-MEMS in Medicine (BAMM) Laboratory, Division of Infectious Diseases, Brigham and Women’s Hospital, Harvard Medical School, MA, USA.

^3^ INCoD - National Brazilian Institute for Digital Convergence/LAPIX, Image Processing and Computer Graphics Lab, Federal University of Santa Catarina, Brazil.

^4^ Demirci Bio-Acoustic-MEMS in Medicine (BAMM) Laboratory, Stanford University School of Medicine, Canary Center at Stanford for Cancer Early Detection, Palo Alto, CA, USA.

^5^ Present address: Biomedical Engineering Department, Faculty of Engineering, Başkent University, Ankara, Turkey.

^6^ 4VisionLab, Master in Applied Computing, University of Itajaí Valley, Brazil.

^7^ CERTI Foundation, Florianopolis, Brazil.

**Running title:** Portable Lensless Wide-field Microscopy Imaging

*Corresponding author: Utkan Demirci, PhD,

Demirci Bio-Acoustic-MEMS in Medicine (BAMM) Laboratory,

Stanford University School of Medicine,

Canary Center at Stanford for Cancer Early Detection,

3155 Porter Drive, Palo Alto, CA 94304, USA

Email: [utkan@stanford.edu](mailto:utkan@stanford.edu)

**Computational Specifications and Analysis**

The experiments described in this paper were conducted on a XPS Intel I7, having distinct computational aspects and execution time. Image acquisition (i) was performed after the sample preparation, and to acquire the LR set (by shifting the light-source) 2 minutes approximately were required. The feature-based registration (ii) is a computationally fast procedure since it is based on key-points detector, and to register a rectangular domain from the LR set, a few seconds were spent. The next procedure, the sub-pixel optimization method (iii), is the most time-expensive procedure, since it is based on the area-matching concept, and the minimization of an error metric of energy is applied to perform sub-pixel optimization. Its execution time can take several minutes to complete a cycle of 200 iterations considering the whole set of LR images. For the both fast alignment and optimization procedures, a rectangular domain area of 1024 × 768 pixels can take at least 5 minutes to complete the estimation of a single HR holographic image. Finally, for the last procedure (iv), the diffraction calculation of a distinct plane is computed in a few seconds considering the whole FOV (3840×2748 pixels).

**Feature-based Image Registration**

In our presented platform, very small displacements (*i.e.*, 1-2mm) of the light source were performed, so therefore the content is almost the same and the scene can be considered approximately planar. Acquisition process is performed under the same wavelength, light intensity and distance between object and detector-plane. Under these controlled circumstances, images can be aligned using both feature-based and area-based (area-matching) methods in sequence. Feature-based methods register images using a set of sparse feature points (named key-points, a minimum of 4 non-collinear points), where the basic principle is finding interesting points and correlate them to solve a matrix of transformations need to wrap any two planes. Feature-based methods are fast to be computed, since the number of key-points is significantly smaller than the structures (pixel, meta-data) commonly used for area-matching approaches. In Figure S1 (left side) a typical feature-based alignment procedure used in our approach is shown. First and second rows indicate distinct shift positions of the light source, where the candidate frame (right) is registered onto a reference image (left), and relevant key-points between them are connected to show similarity. High-lighted areas in the feature-based registration method correspond to the key-points, where displacement vectors for the matrix are computed (lines in red color).

On the other hand, area-based methods need some error metric to describe how well the images match, by selecting the parameters for the matrix-matching that optimizes the quality of the registration.

**Sub-pixel Optimization using ACO_ℜ_**

The essence of traditional ACO algorithms for discrete optimization problems is proceeding an iterative construction of solutions based on the probabilistic selection of candidate solutions, biased by the pheromone concept [1]. The idea of pheromone is used to indicate the decision variables in **S**, whose quality is better to solve the optimization problem.This information of locality is then passed to the other ants, to indicate promising regions in the search-space and to visit neighborhood solutions. To avoid too fast-convergence, pheromone evaporation is used where good solutions found earlier are used to increase the probability of the search. The algorithm works in an incremental manner, and has its variable solutions determined by the own problem formulation: discrete solution components are probabilistically chosen by a discrete function at each interaction, and an error metric of fitness is updated for each new candidate solutions.

Optimization for continuous domains containing real variables (ACO_ℜ_), on the other hand, uses a kernel based on Gaussian probability density functions (PDF), and instead choose a discrete component, an ant samples the own density function [1]. This is done by probabilistically sampling solutions and its update has as objective to increase or decrease the values associated with good/promising solutions and bad ones, respectively. Density function in ACO_ℜ_ is associated with a cumulative distribution function, used to produce uniformly distributed real numbers for decision variables. To prevent the problem of disjoint areas in the search-space, the PDF is based on a Gaussian kernel function computed from a set of 1-dimensional Gaussians. The PDF then weights each Gaussian function into a non-linear function for each decision variable. An advantage of Gaussian kernel PDFs is the reduction of dimensionality (one PDF for each decision variable), allowing a reasonably easy sampling and flexibility when compared to a set of single Gaussian functions.

For continuous domains, pheromone information is not represented like traditional ACO model (pheromone information is stored as a table), since the choice is not limited to a finite set of values (but real values). To solve this limitation, the strategy adopted is to update pheromone based on good candidate solutions (like in discrete ACO), but instead of eliminate current solutions, the ACO_ℜ_ maintain a history of solutions and the pheromone evaporation is associated with the oldest solutions in the archive **T**. ACO_ℜ_ stores in **T** the values corresponding to ***n*** decision variables and ***s*** solution scores, associated with the objective function **f(s*_n_*)**. Differently from regular ACO approaches or ACO population-based algorithms, where solutions update pheromone, in ACO_ℜ_ pheromone is applied to generate PDFs in a dynamic manner [1].

From a technical point of view, the ACO_ℜ_ is denoted as an excellent framework for the convergence and optimization of continuous decision variables, seeking for the minimization of an error metric or objective function. Similarly, variational models present the energy minimization postulated as a set of premises to solve a model, and by minimizing the energy (*i.e.*, ***E → 0***), better is the approximation to this model, where every premise should be minimally satisfied. In general, the aforementioned premises are modeled by penalizer terms of the model, and energy minimization approaches have been successively applied over many image processing areas, such as image segmentation, restoration and filtering, denoising [2], and also for super-resolution in video sequences [3, 4].

In our approach, the minimization of a functional of energy is mainly determined by the sharpness measure, computed from the LR set when an optimized sub-pixel registration is obtained. Sharpness is one of the most important aspects of photographic quality, representing the level of details an imaging device can reproduce. The use of a sharpness measure as an indicator of holographic photonic quality is demonstrated in Figure S1 – right side, where two distinct candidate HR solutions are presented. For each HR solution, a scalar value corresponding to its quality is obtained, according to Eq.1 in the manuscript. Candidate solution shown in the bottom side presents a better index of energy, increasing the sharpness level and propagation of holographic information. Over the literature several methods have been proposed to the measure sharpness level of an image, and an extensive validation of these approaches designed to compute a general overall has been performed [5]. This evaluation indicates Laplace-based operators as the best category of methods for focus measure, and to operate at normal environmental conditions. Under varied or uncontrolled conditions of illumination, or using distinct acquisition devices from different manufactures, it may be difficult to determine the best general performance for focus methods.

In our Supplementary Video 6, both feature-based and sub-pixel optimization approaches are illustrated for the reconstruction of holographic sperm samples, where computational details and holographic resolution improvements can be verified.

**Diffraction Calculation**

Diffraction is a very important aspect in holography, being practically used for a wide-ranging of optics fields, revealing important properties of diffracting objects. Diffraction in Fourier optics is classified into convolution methods, where a kernel is used to convolve the input signal, or in the frequency domain by means of a direct Fourier transform. The Angular Spectrum Method (ASM) is one of the convolution approaches for diffraction calculation, designed to operate at short-distance between object and detector planes [6]. ASM is based on the propagation of a set of infinite plane-waves, and can be expressed by the equation below:

$u\left(x,y\right)=\int\int_{-\infty}^{+\infty} A\left(f_{x},f_{y}\right)H\left(f_{x},f_{y}\right)\text{exp}\left(i2\pi\left(f_{x}x+f_{y}y\right)\right)df_{x}df_{y}$. (S1)

**A(f_x_, f_y_)** is a Fourier transformation to ***u(x,y)***, and **f_x_** and **f_y_** correspond to the spatial frequencies. The response impulse function is a transfer function is defined by **H**, and can be estimated using a Fast Fourier Transform, associated with its inverse part:

$u\left(m,n\right)=FFT^{-1}\left[FFT\left[u\left(m,n\right)\right]H\left(m_{1},n_{1}\right)\right]$, (S2)

The discrete **f_x_** and **f_y_** frequencies are related to the destination source (**f_x_**,**f_y_**)=(***m_1_*** *∆***f_x_*, n_1_****∆***f_y_**), where ***m_1_, n_1_*** are integer indices on this plane, having *∆***f_x_** and *∆***f_y_** on the frequency domain as the sampling pitches of the electronic device. The diffractive distance between the source plane ***u(x_1_,y_1_)*** and the destination plane ***u(x,y)*** (detector-plane) is used as a parameter of the numerical diffraction calculation. We used *z×λ* to compute the distance between the object and the detector plane, where image is formed with a wavelength of the light-source *λ*. For HR holograms, a scale factor *k* is applied to up-sampling the distance *z* and make diffraction equivalent to the LR and HR images.

The transfer function **H** is given by:

$H\left(f_{x},f_{y}\right)=\text{exp}\left(iz\sqrt{k^{2}-4\pi^{2}\left(f_{x}^{2}+f_{y}^{2}\right)}\right)$. (S3)

where ***k=2π /λ*** and ***z*** are used as the wave-number and the distance between object and detector plane. From S3, phase and amplitude can be obtained by θ*(x,y)=* tan^-1^[ℑ*(x,y) /* ℜ*(x,y)*] and r*(x,y)=*[ℜ*(x,y)*^2^+ℑ*(x,y)*^2^]*^1/2^*, respectively from the real (ℜ) and imaginary (ℑ) parts. A multi-dimensional signal is also presented by combining both ℜ and ℑ in individual channels.

**Holographic Signal Resolution**

Increase of spatial resolution can be obtained from a set of LR observations of the scene when the number of frames is sufficient, and its content is shifted by sup-pixel proportions. Under these conditions, it is possible to recombine phase to recover some level of detail into a single HR image, with a large density of pixels [7]. The use of multiple frames for sub-pixel computation applied to more than 50 reference images has been previously used to achieve a higher spatial resolution [8]. The number of images, however, depends of the quality of sub-pixel shifts to capture sufficient information.

The obtained results demonstrate the effectiveness of the presented holographic platform in terms of hardware and software parts. Holographic information, or in other terms, the propagation of high-frequency fringes determine the level of details to be recovered the numerical diffraction method. In Figure S2, the USAF1951 resolution chart imaged by the presented holographic platform is illustrated, where a single shot frame (LR) and a reconstructed frame (HR) from the LR set are presented. The Prewitt compass from a single shot LR and HR frame are also shown in the middle. Similarly to the experimental results demonstrated in the manuscript, holographic signals were measured as shown by the green and red lines, where pixel intensities were compared in the graph illustrated at the top of Figure S2. Two main aspects can be observed based on this analysis: (i) improvement of the periodical propagation of the holographic information, and (ii) spatial and temporal noise suppression by summation in the background areas. Holographic signal propagation was recovered in the HR image as shown by the graph plotted (from the left side to the middle), where a large number of wave cycles can be observed. Noise suppression is verified from the middle to the right part of the same graph, where the frequency becomes invariant and tends to be preserved, and indicating strong background areas recovered from the LR set.

Additionally, the USAF1951 resolution chart is also presented to determine the power of resolution improvement, as shown in Figure S3. For this purpose, a single shot frame (LR) is compared against 3×3 shifts (9 frames) and also 7×7 (49 images). The obtained results show improvement of the resolution for the multiple 7×7 frames (group number 8 / Element 3 (1.55 µm resolution)) compared to 3×3 frames (group number 7 / Element 6 (2.19 µm resolution)) and a single frame (group number 7 / Element 4 (2.76 µm resolution)). Obtained results of the presented holographic platform applied to sperm samples are also shown in the Figure 5 of the manuscript.

**
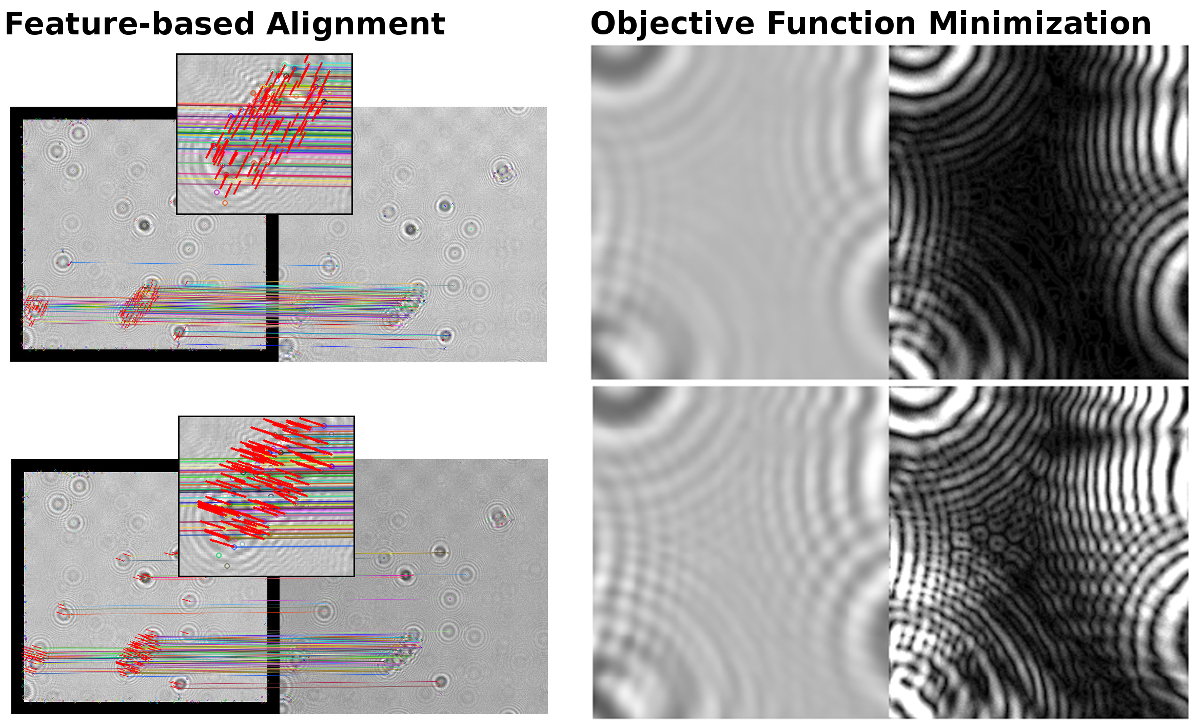
**

**Figure S1 Fast alignment procedure based on features and the sub-pixel optimization procedure.** At the left side, registration is based on a set of key-points, where a reference frame is used to align the LR set. Reference and candidate frames are then connected (color lines shown at the left side), and for each one a displacement vector (in red color) indicates the differences in length and orientation between them. At the right side, the sub-pixel optimization procedure for two candidate solutions (top and bottom) are demonstrated. The initial sup-pixel alignment (top) is compared to the result obtained after some iterations (bottom), illustrating the minimization of the energy to increase sharpness and high-frequency propagation, respectively.


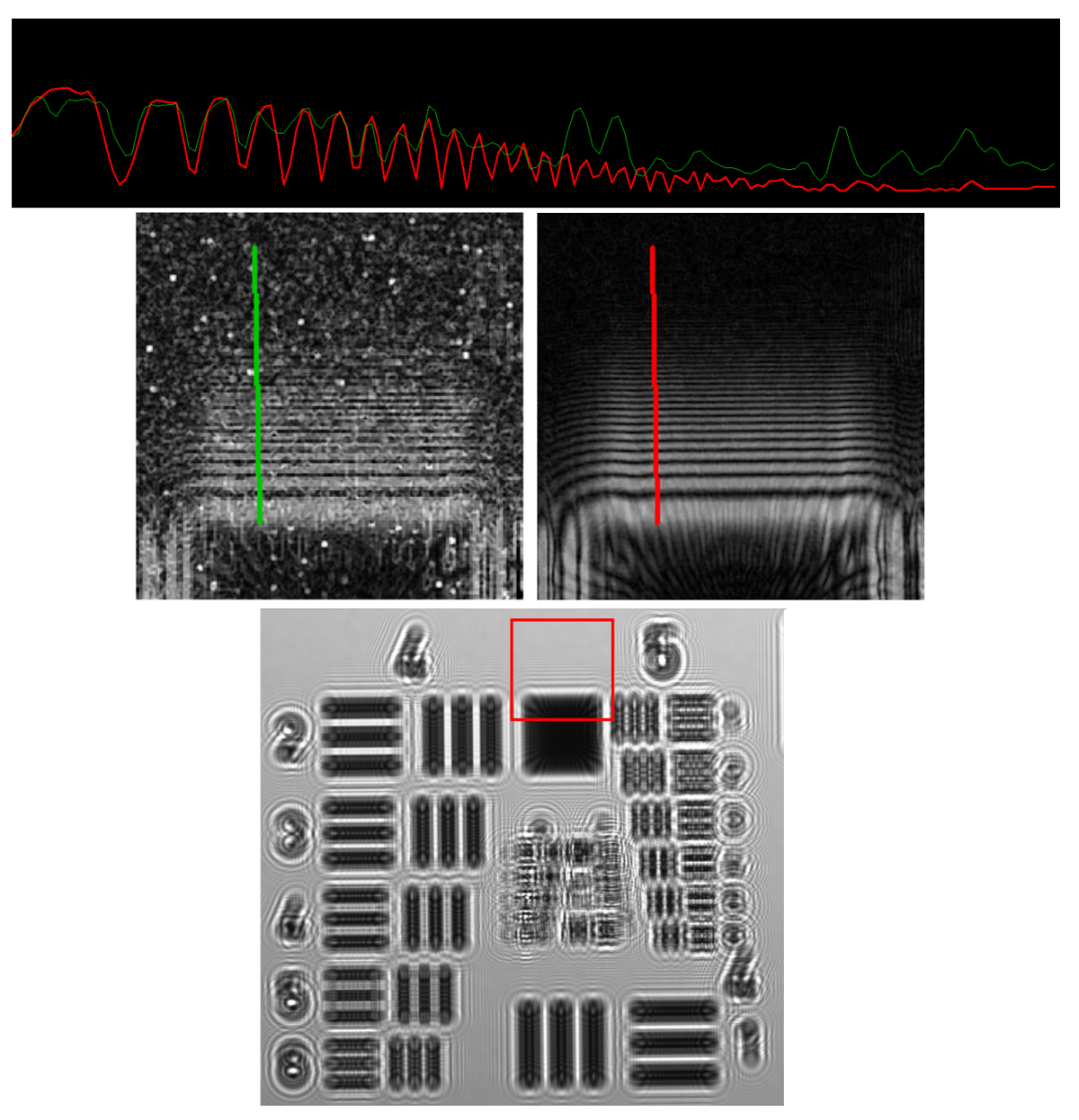


**Figure S2 Holographic signal of USAF1951 Resolution Chart.** Middle, left and right sides correspond to a LR and a HR reconstructed signal, respectively. Measurements of holographic intensity were performed as shown at the top, where LR (green) and HR (red) lines are presented.


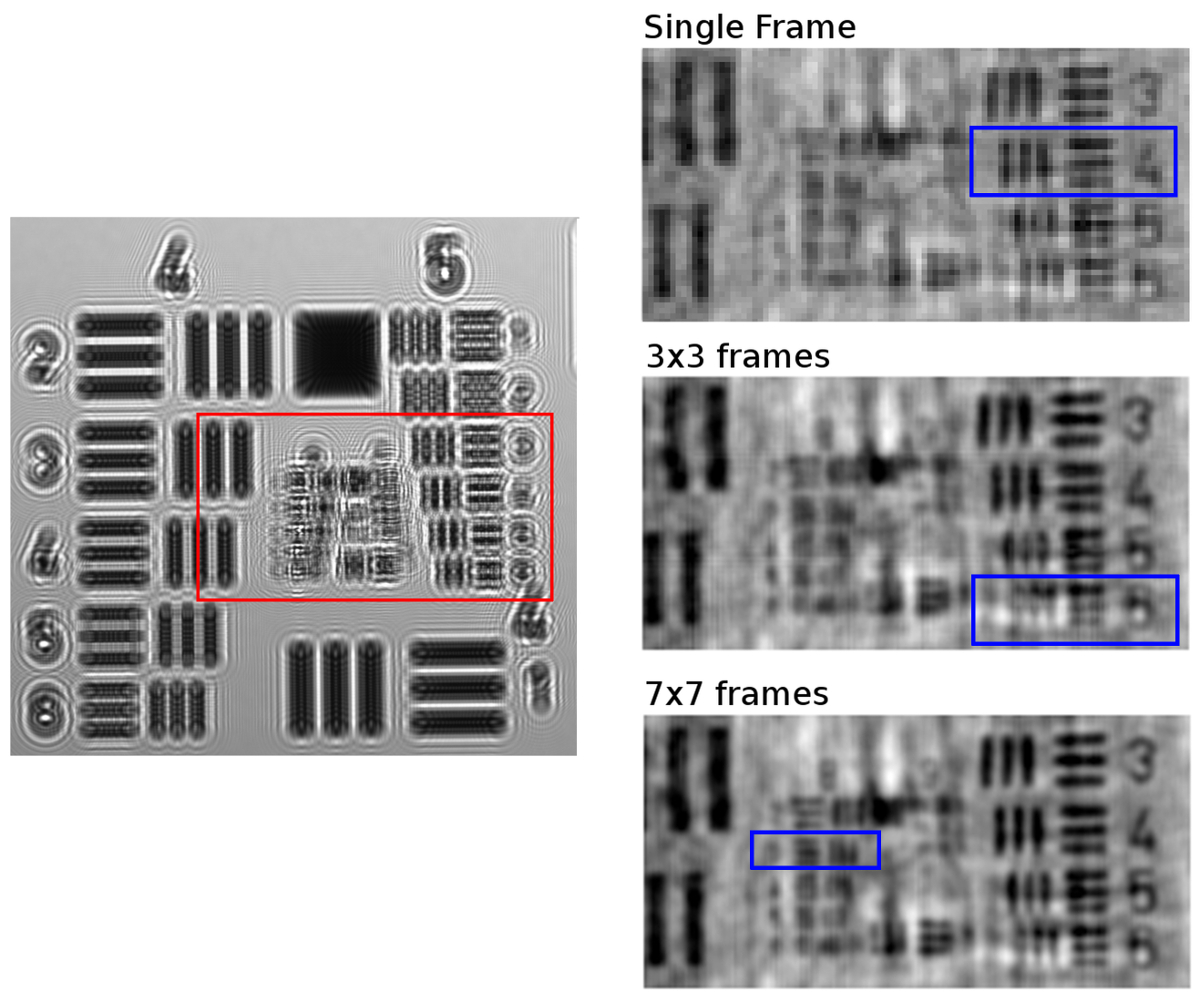


**Figure S3 Diffraction calculation for USAF Resolution Chart**. The selected holographic area is presented after decoded by ASM using a single shot frame, arbitraryshifts of 3×3 (9 frames) and 7×7 (49 frames). Blue dashed-lines show the minimum resolution features monitored with corresponding method.

| **Approach** | **Scheme / light-source shifts** | **Detector Pixel-size (µm)** | **Resolution**  **(µm)** | **FOV (mm^2^)** | **Reference** |
| --- | --- | --- | --- | --- | --- |
| Ozcan`s Group | Precise with an array of 23 LED`s | 1.67, 2.4 | ~1 | ~24 | [9][10] |
| Ferraro`s Group | Spatial diffraction 2D grating | 7.60 | NA | NA | [11] |
| Presented Approach | Arbitrary, automatic registration | 1.67 | ~1.55 | ~30 | Our |

**Supplementary Table S1.** Comparison of Portable Digital In-line Holography Platforms based on sub-pixel computation.

**
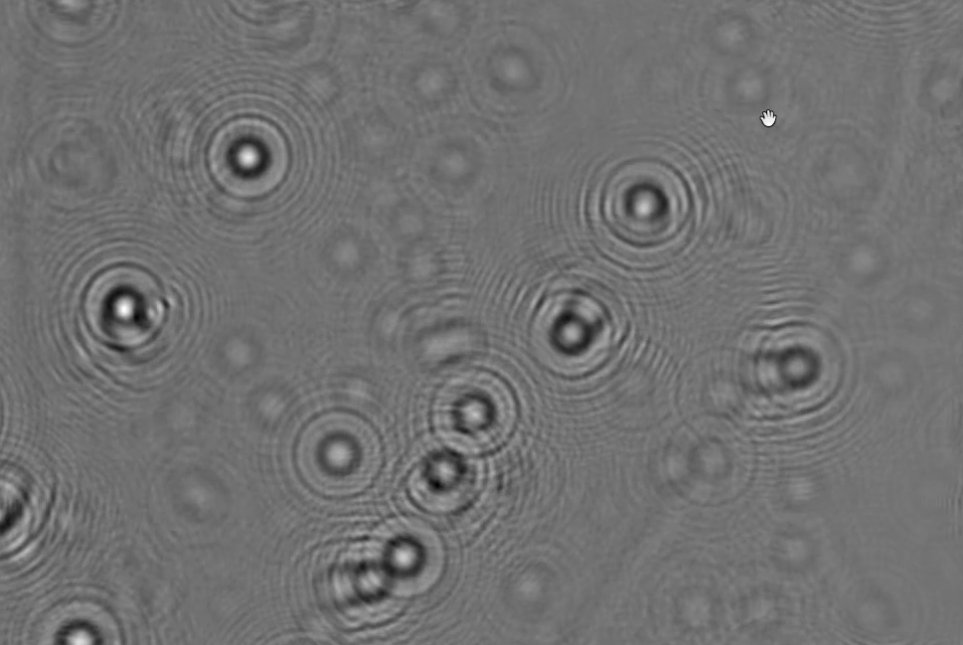
**

**Supplementary Video 1-5.** Diffraction of a sperm sample hologram.The real (ℜ), imaginary (*ℑ*), phase (*θ), amplitude (r)* and composition (*ℜ+ℑ*) of the signals were used, respectively, to demonstrate the numerical diffraction process at different z-axis object-planes.


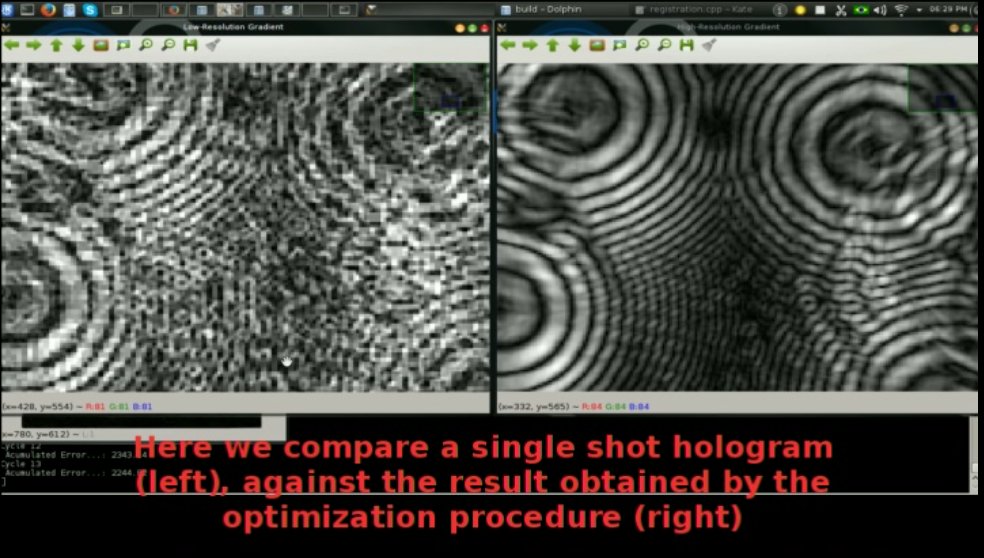


**Supplementary Video 6.** Computational methods to create a HR single image from a set of 49 LR holograms. After selecting a region of interest to be analyzed, a rapid registration procedure aligns the holograms into the same planar domain, then an optimization procedure is applied to increase holographic signal based on the sharpness level.

**REFERENCES**

[1] K. Socha, M. Dorigo, Ant colony optimization for continuous domains, European Journal of Operational Research 185 (3) (2008) 1155–1173.doi:http://dx.doi.org/10.1016/j.ejor.2006.06.046.

[2] T. Brox, From Pixels to Regions: Partial Differential Equations in Image Analysis, Ph.D. thesis,Mathematical Image Analysis Group, Department of Mathematics and Computer Science, Saarland University, Germany (Apr. 2005).

[3] D. Mitzel, T. Pock, T. Schoenemann, D. Cremers, Video super resolution using duality based tv-l1 optical flow, in: Proceedings of the 31st DAGM Symposium on Pattern Recognition, Springer-Verlag, Berlin, Heidelberg, 2009, pp. 432–441.

[4] Y. Mochizuki, Y. Kameda, A. Imiya, T. Sakai, T. Imaizumi, Variational method for super-resolution optical flow, Signal Processing 91 (7) (2011) 1535 – 1567.

[5] S. Pertuz, D. Puig, M. A. Garcia, Analysis of focus measure operators for shape-from-focus, Pattern Recognition 46 (5) (2013) 1415 – 1432.doi:http://dx.doi.org/10.1016/j.patcog.2012.11.011.

[6] T. Shimobaba, J. Weng, T. Sakurai, N. Okada, T. Nishitsuji, N. Takada, A. Shiraki, N. Masuda, T. Ito, Computational wave optics library for C++: CWO++ library, j-COMP-PHYS-COMM 183 (5) (2012) 1124–1138. doi:http://dx.doi.org/10.1016/j.cpc.2011.12.027.

[7] Simpkins, R.L. Stevenson, "An Introduction to Super-Resolution Imaging." Mathematical Optics: Classical, Quantum, and Computational Methods, Ed. V. Lakshminarayanan, M. Calvo, and T. Alieva. CRC Press, 2012.539-564.

[8] A Greenbaum, ALuo, AKhademhosseinieh, Bahar A, Ting-Wei, ACoskun, Ahmet F, AOzcan. T Increased space-bandwidth product in pixel super-resolved lensfree on-chip microscopy.JScientificreports.V 3. 2013.

[9] O. Mudanyali, D. Tseng, C. Oh, S. O. Isikman, I. Sencan, W. Bishara, C. Oztoprak, S. Seo, B. Khademhosseini, A. Ozcan, Compact, light-weight and cost-effective microscope based on lensless incoherent holography for telemedicine applications, Lab Chip 10 (2010) 1417–1428. doi:10.1039/C000453G.

[10] W. Bishara, T.-W.Su, A. F. Coskun, A. Ozcan, Lensfree on-chip microscopy over a wide field-of-view using pixel super-resolution, Opt. Express 18 (11) (2010) 11181–11191. doi:10.1364/OE.18.011181.

[11] M. Paturzo, F. Merola, S. Grilli, S. De Nicola, A. Finizio, and P. Ferraro, "Super-resolution in digital holography by a two-dimensional dynamic phase grating," Opt. Express 16, 17107-17118 (2008).
